# NMR-STAR: comprehensive ontology for representing, archiving and exchanging data from nuclear magnetic resonance spectroscopic experiments

**DOI:** 10.1007/s10858-018-0220-3

**Published:** 2018-12-22

**Authors:** Eldon L. Ulrich, Kumaran Baskaran, Hesam Dashti, Yannis E. Ioannidis, Miron Livny, Pedro R. Romero, Dimitri Maziuk, Jonathan R. Wedell, Hongyang Yao, Hamid R. Eghbalnia, Jeffrey C. Hoch, John L. Markley

**Affiliations:** 1 Biochemistry Department, University of Wisconsin-Madison, Madison, WI 53706 USA; 2 Department of Computer Sciences, University of Wisconsin-Madison, Madison, WI 53706 USA; 3 ATHENA Research and Innovation Center, Athens, Greece; 4 Department of Molecular Biology and Biophysics, UConn Health, 263 Farmington Avenue, Farmington, CT 06030 USA

**Keywords:** Archival data, Biomolecules, Experimental conditions, Experiment description, Data specification, NMR-STAR, Nuclear magnetic resonance spectroscopy, Ontology, Python, R environment, Visualization of data

## Abstract

The growth of the biological nuclear magnetic resonance (NMR) field and the development of new experimental technology have mandated the revision and enlargement of the NMR-STAR ontology used to represent experiments, spectral and derived data, and supporting metadata. We present here a brief description of the NMR-STAR ontology and software tools for manipulating NMR-STAR data files, editing the files, extracting selected data, and creating data visualizations. Detailed information on these is accessible from the links provided.

## Introduction

NMR-STAR is the archival format used by the Biological Nuclear Magnetic Resonance data Bank (BMRB), the international repository of biomolecular NMR data (Ulrich et al. 2008) and an archive of the Worldwide Protein Data Bank (wwPDB 2018). NMR-STAR is available as input and/or output by several software packages that deal with the harvesting and processing of biomolecular data [CCPN (Vranken et al. 2005), NMRView (Johnson 2004), TALOS (Cornilescu et al. 1999), NMRFAM-SPARKY (Lee et al. 2015), PINE (Bahrami et al. 2009), ARECA (Dashti et al. 2016), PONDEROSA (Lee et al. 2011), Integrative NMR (Lee et al. 2016), CSI (Hafsa et al. 2015), NMRFx^1^, RCI (Berjanskii and Wishart 2007), ABACUS (Grishaev et al. 2005), relax (d’Auvergne et al. 2008; d’Auvergne and Gooley 2008), and PDBstat (Tejero et al. 2013)] and with chemical shift prediction [SHIFTX2 (Han et al. 2011) and SHIFTS (Xu and Case 2002)]. NMR-STAR is also used as a data exchange format by the NMRbox project (Maciejewski et al. 2017).

**Fig. 1:**
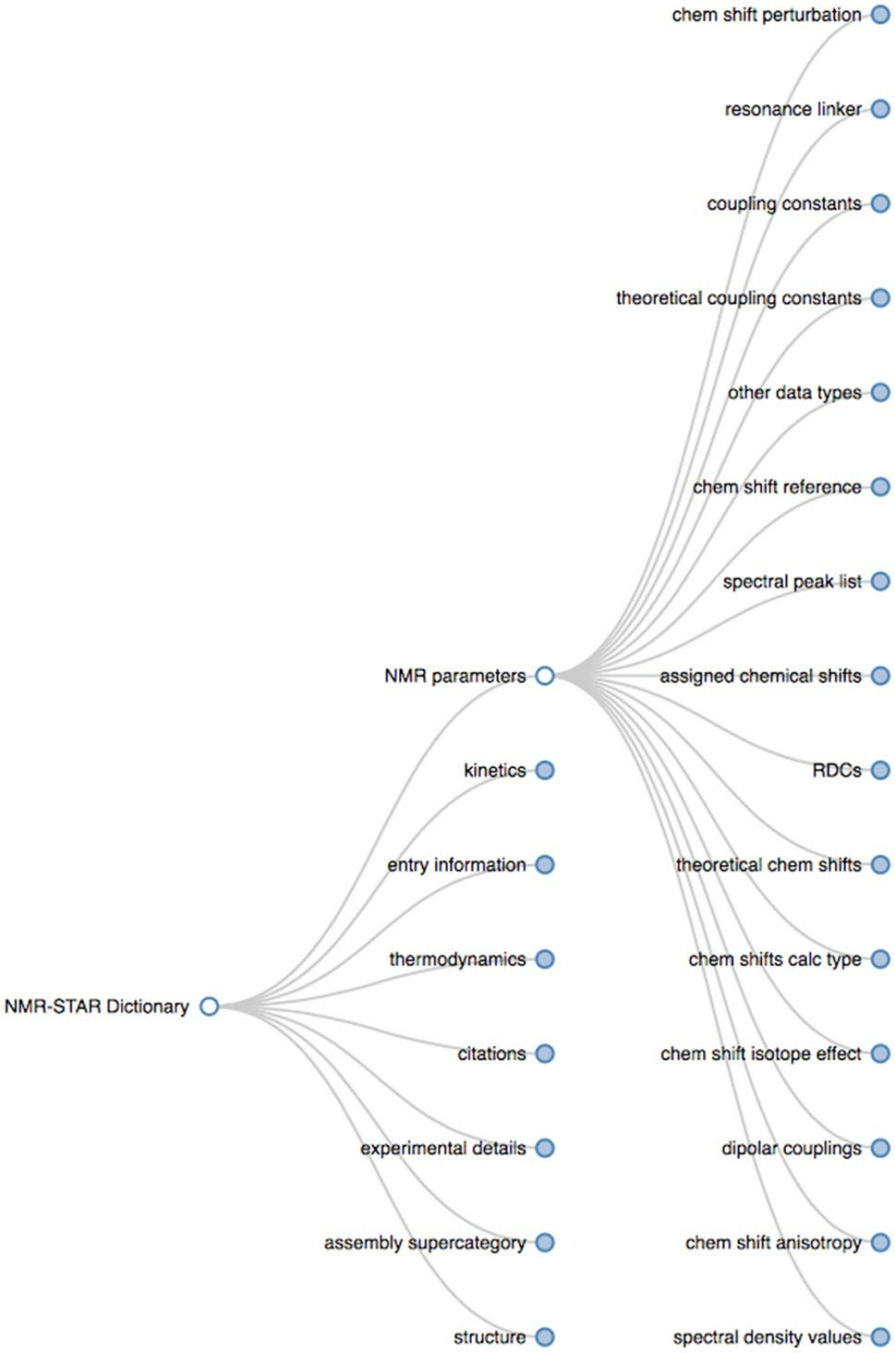
First two tiers in the NMR-STAR ontology for BMRB. The STAR dictionary tree is an interactive tool for inspecting the NMR-STAR ontology and assessing the most appropriate construct for the user’s application. The NMR-STAR ontology is under constant evolution with community feedback to reflect advancements in NMR techniques. An interactive version is available from the BMRB website with tools that enable searching by category or tag

We describe here the NMR-STAR ontology and associated software tools that facilitate its use. The NMR-STAR v3.2 ontology^2^ provides an extensive controlled vocabulary for the description of NMR spectroscopic studies of biological systems. The ontology includes the description of experiments, the data generated, and the derived results such as molecular structures, dynamics, and functional properties. NMR-STAR v3.2 is constructed along the lines of an object/relational model using a subset of the Self-defining Text Archival and Retrieval (STAR) specification (Hall and Spadaccini 1994). Full documentation on the rules and conventions for constructing valid NMR-STAR formatted files is available from the BMRB website.^3^^,^^4^ The data in a BMRB entry as defined by the NMR-STAR ontology are organized in natural objects such as citations, molecular entities, samples, software applications, NMR experiments, and experimental data sets of various kinds^5^ (see Fig. 1). However, the data also are organized in tables within the objects and are referentially linked using primary and foreign keys forming an entity/relational model, as well. The NMR-STAR ontology, therefore, can be used to create a relational schema and database. The data from multiple entries can be easily organized as tables for loading into a relational database constructed from the NMR-STAR ontology.

While NMR-STAR is intended to cover the vast majority of biomolecular NMR information, data that are massive and more appropriately stored in binary or other formats (for example, time-domain and processed multidimensional NMR spectral data) or instrument specific (pulse sequence files) are modeled as external files. Archiving and exchanging these kinds of data is very important, but files of these kinds can be referenced and associated with an NMR-STAR file without being incorporated into that file.

The NMR-STAR ontology was first released for use as a deposition, archival, and data exchange format in 1996 (Ulrich et al. 1996). At that time, the STAR format was chosen over alternatives, such as Abstract Syntax Notation One (ASN.1), standard generalized markup language (SGML), and extensible markup language (XML), because STAR met the criteria for extensibility while at the same time the format addressed the needs for a developing and expanding scientific field: combined machine and human readability, efficient editing by existing tools, and ease of mapping to relational database technology. The original NMR-STAR version 1.0 accommodated a limited number of experimental data (assigned chemical shifts, coupling constants, peak lists, and relaxation parameters). With input from many scientists in the NMR, X-ray crystallography, and computer science communities, the ontology has expanded to include over 90 data category groups containing a total of more than 300 data categories and over 6500 data item tags. Because the Crystallography Information File (CIF) format used by the small molecule crystallography community and the mmCIF format used by the Protein Data Bank (PDB) are subsets of STAR, NMR-STAR communicates easily with these repositories. Extensible markup language (XML) and resource description framework (RDF) versions of NMR-STAR have been developed and are available from PDBj-BMRB (Yokochi et al. 2016).

The ontology has evolved over time with input from the user community. The entire BMRB archive has been upgraded to NMR-STAR v3.2 for consistency with legacy data. NMR-STAR v3.2 incorporates a variety of experimental data (e.g., coupling constants, heteronuclear NOEs, T1/R1, T2/R2, and autorelaxation and dipole–dipole cross correlation parameters). In NMR-STAR v3.2 ‘molecular interactions’ tags have been redefined as ‘chemical shift perturbation’ to match the common terminology used by the NMR community. The ontology has been expanded to capture additional parameters derived from experiments as well as data processing workflows.

The NMR exchange format (NEF) (Gutmanas et al. 2015) utilizes the STAR format and defines an ontology simpler than NMR-STAR for the purpose of facilitating data exchange between structural NMR software packages. The wwPDB has agreed to accept both NEF and NMR-STAR as deposition formats for structural restraints. The BMRB has adopted NEF as a subset of NMR-STAR and has developed software to convert restraints in NEF to the archival NMR-STAR format that serves as a more comprehensive exchange format for restraints, in that it handles restraint types not covered by NEF (including ambiguous restraints between subunits, restraints to ligands, residual dipolar couplings, paramagnetic relaxation enhancement, and restraints derived from cross-linking or SAXS). Because NEF utilizes non-unique atom designators rather than standard IUPAC, NEF can be converted to NMR-STAR, but NMR-STAR cannot be converted uniquely back to NEF. For that reason, the wwPDB stores the original NEF from depositions and requires NEF depositions to include coordinates in mmCIF format with a clear mapping from the atom names used in the NEF files to the IUPAC format used by mmCIF/NMR-STAR. In response to suggestions from the NEF team, NMR-STAR V3.2 supports views of spectral peak list information that merge data from four tables into one more-readable table. The BMRB retains the four-table format for purposes of database management.

Users can retrieve data from the NMR-STAR archive by means of a variety of query interfaces available on the BMRB website (http://www.bmrb.wisc.edu/). Queries can be based either on the NMR-STAR data model or on information extracted from BMRB entries by means of the NMR-STAR dictionary. PyNMRSTAR is a library for interacting with NMR-STAR files in the Python language by reading files, modifying them, and writing them out.^6^ The PyNMRSTAR library is used by a variety of tools developed by the BMRB or external groups for operating with BMRB files. The RBMRB software package provides access to BMRB data in the R environment.^7^ One can use built-in functions in RBMRB to visualize data mined from BMRB and to simulate spectra. Third-party software developers have provided Perl script for converting NMR-STAR to NMRPipe.^8^ and Python parsers for NMR-STAR that support interfacing with BMRB (Smelter et al. 2017).^9^

## Discussion

Conceptually, a BMRB entry can be viewed as a set of linked objects (saveframes) connected by the STAR saveframe pointer method (Hall and Cook 1995), by the use of integer object identifiers, and by relational primary and foreign keys. The NMR-STAR data model, as a relational schema, is highly denormalized with redundant information present across many tag categories or tables. There also exist multiple paths for linking experimental data with the experiments, samples, sample conditions, and other metadata. This has allowed data derived from the literature to be represented in the relational database, even when the information required to link metadata to experimental data is not always complete. While NMR-STAR is feature-rich, the format is flexible and allows for lightweight use. For example, subsets of the full NMR-STAR ontology are utilized in workflows involving data exchange. The combination of rich representation and lightweight application provides application developers an evolutionary path to data exchange while at the same time it mitigates the N^2^ format-to-format translation problems. For ease of on-line searching, saveframes and tags are organized into categories and sub-categories.^10^ Each tag is fully described with data type and mandatory status defined.

Information encoded in the NMR-STAR ontology, supplemented by interface specification files, drives the deposition interface at BMRB. Templates are available from the BMRB website for generating and validating NMR-STAR files for a variety of NMR data, including assigned chemical shifts, coupling constants, hydrogen exchange data, and a variety of relaxation data. A number of third party software applications read and write NMR-STAR compliant files that are ready for deposition. Software tools are available for converting data from various software packages to NMR-STAR or NEF. The most popular are PDBStat (Tejero et al. 2013) and STARch (STAR conversion handler).^11^ STARch converts data tables in NMR-STAR 2.0 to 3.0. Depositors of NMR data to wwPDB archives are encouraged to use these or similar tools to convert their data into NMR-STAR (or NEF) prior to submission.

## Data Availability

Data and software tools are available from the BMRB website (http://www.bmrb.wisc.edu/). The interactive version of the NMR-STAR ontology is available from: http://www.bmrb.wisc.edu/dictionary/interactive/dict_tree.shtml.

## Abbreviations

ASN.1: Abstract Syntax Notation One
BMRB: BioMagResBank or Biological Magnetic Resonance data Bank
CIF: Crystallographic Information File
NMR: Nuclear magnetic resonance
PDB: Protein Data Bank
PDBj: Protein Data Bank of Japan
RDF: Resource description framework
SGML: Standard generalized markup language
STAR: Self-defining text archival and retrieval
XML: Extensible markup language

## Footnotes

1 NMRFx, https://nmrfx.org/.

2 NMR-STAR v3.2 ontology, http://www.bmrb.wisc.edu/formats.html.

3 BMRB dictionary, http://www.bmrb.wisc.edu/dictionary/.

4 NMR-STAR 3.2 dictionary, http://svn.bmrb.wisc.edu/svn/nmr-star-dictionary/bmrb_only_files/adit_input/NMR-STAR.dic.

5 Interactive version of the NMR-STAR ontology, http://www.bmrb.wisc.edu/dictionary/interactive/dict_tree.shtml.

6 Library for interacting with NMR-STAR files in the Python lauguage: PyNMRSTAR, https://github.com/uwbmrb/PyNMRSTAR.

7 Access to BMRB data in the R environment: RBMRB, https://cran.r-project.org/package=RBMRB.

8 Perl script for converting NMR-STAR to NMRPipe, https://qa.nmrwiki.org/question/335/spectra-from-bmrb-chemical-shifts.

9 A library for parsing data in NMR-STAR format, https://github.com/mattfenwick/NMRPyStar.

10 Saveframe and tag organization in NMR-STAR, http://www.bmrb.wisc.edu/dictionary/supergrp.php.

11 STAR conversion handler (STARch) http://www.bmrb.wisc.edu/software/starch/.
